# POLICY SERIES: HEALTH AND AGING POLICY FELLOWS: LINKING POLICY AND PRACTICE TO BENEFIT OLDER PEOPLE

**DOI:** 10.1093/geroni/igad104.0022

**Published:** 2023-12-21

**Authors:** Maureen Henry, Emily Franzosa

## Abstract

The Health and Aging Policy Fellows program was founded in 2008 after its founders observed a dearth of experts with overlapping experience in policy and aging. The program is creating a growing cadre of leaders to improve the health of older adults by influencing policy at the state and national levels that is joined through an alumni network of more than 150 fellows. The fellowship’s goal is to improve the lives of older people across life domains. Past fellows serve as staff members in Congress, in State and Federal Agencies, and in the private sector. The fellowship has helped to launch many policy-focused careers. In this seminar, current and past fellows from medicine, research, social work, and law will share their experiences before, during, and after their fellowship. They will describe what they learned through the training and a placement in a full- or part-time policy role, how viewing health and aging through a policy perspective changed their understanding of systems, and how they think the fellowship will enable them to influence policy to improve the lives of older people in the future. Fellows will also highlight ongoing policy challenges that stop or slow progress toward achieving the goal.

